# Similar but different: High prevalence of synesthesia in autonomous sensory meridian response (ASMR)

**DOI:** 10.3389/fpsyg.2022.990565

**Published:** 2022-09-29

**Authors:** Giulia L. Poerio, Manami Ueda, Hirohito M. Kondo

**Affiliations:** ^1^School of Psychology, University of Sussex, Falmer, United Kingdom; ^2^Department of Psychology, University of Essex, Colchester, United Kingdom; ^3^School of Psychology, Chukyo University, Nagoya, Japan

**Keywords:** autonomous sensory meridian response, synesthesia, multisensory, cross-modal correspondence, emotion

## Abstract

Autonomous sensory meridian response (ASMR) is a complex sensory-emotional experience characterized by pleasant tingling sensations initiating at the scalp. ASMR is triggered in some people (called ASMR-responders) by stimuli including whispering, personal attention, and crisp sounds (termed ASMR triggers). Since its inception, ASMR has been likened to synesthesia, but convincing empirical data directly linking ASMR with synesthesia is lacking. In this study, we examined whether the prevalence of synesthesia is indeed significantly higher in ASMR-responders than non-responders. A sample of working adults and students (*N* = 648) were surveyed about their experience with ASMR and common types of synesthesia. The proportion of synesthetes who were classified as ASMR-responders was 52%, whereas 22% of ASMR-responders were also synesthetes. These results suggest that: (1) over half of those identifying as synesthetes also experience ASMR, and (2) that synesthesia is up to four times as common among ASMR-responders as among non-responders (22% vs. 5%). Findings also suggest a prevalence rate for ASMR of approximately 20%. Overall, the co-occurrence of ASMR and synesthesia lends empirical support to the idea that ASMR may be driven by synesthetic mechanisms, but future research would benefit from examining how ASMR and synesthesia are different, as well as similar.

## Introduction

Autonomous sensory meridian response (ASMR) is a pseudo-scientific term used to describe a complex sensory-emotional experience characterized by pleasant tingling sensations initiating in the scalp. ASMR is reliably triggered in some people (called ASMR-responders) by stimuli such as whispering, soft touch, personal attention, crisp sounds, and slow hand movements (termed ASMR triggers) (Poerio, 2020). Since its discovery, ASMR has been likened to synesthesia, a condition in which a stimulus in one sense elicits a concurrent percept in a second sense (e.g., Ward, 2013). For instance, ASMR has been hypothesized to be a synesthetic-type experience (Barratt and Davis, 2015; Poerio, 2016). Despite this increasingly popular notion, convincing empirical data directly linking ASMR with synesthesia is lacking. Connections between the two phenomena are typically inferred by reference to their similar phenomenology and neurocognitive profiles, rather than by actual investigation. In this study, we examine, for the first time, whether the prevalence of synesthesia is indeed significantly higher in ASMR-responders compared to non-responders. Since synesthetic experiences tend to co-occur (Barnett et al., 2008; Chun and Hupé, 2013), such an association would lend empirical support to the idea that ASMR may be driven by synesthetic mechanisms.

The idea that ASMR is a form of synesthesia is important, because if they are closely related, then synesthetic accounts can be leveraged to explain how and why ASMR arises and develops. If incorrect, however, it may be more prudent for ASMR research to focus on parallels with other complex, but not traditionally considered synesthetic, emotional experiences, such as music-induced chills, to understand its underlying mechanisms. What evidence is there that ASMR and synesthesia are associated? For the most part, the evidence provides indirect, rather than direct, support for this association by emphasizing their ostensible similarities. Nevertheless, these insights make a good argument for why we might predict the incidence of synesthesia to be greater in ASMR-responders. We review that evidence below.

Autonomous sensory meridian response, like synesthesia, is a non-universal, cross-modal, subjective experience that seems to have largely developmental origins. ASMR-responders, like synesthetes, are often surprised when they discover that ASMR is neither universal nor unique (Poerio, 2016). Specifically, ASMR-responders tend to be either: (1) surprised that their perceptual experience is different and somewhat remarkable, believing ASMR sensations to be shared by everyone or (2) relieved that ASMR is also experienced by others, i.e., comforted that they are not somehow “weird.” Such a reaction seems to parallel experiences of synesthetes (Ward, 2013), but is also likely to be true for other anomalous or non-universal phenomena, e.g., aphantasia, sleep paralysis. Descriptive data indicate that both typically emerge in childhood with synesthetes and ASMR-responders reporting their experiences from an early age and often for as long as they can remember (Simner and Bain, 2013; Barratt and Davis, 2015; Poerio et al., 2018). Again, childhood onset is not unique to either ASMR or synesthesia, but does suggest a common, and perhaps shared, developmental origin.

If we assume at a very basic level, that synesthesia requires a consistent inducer-concurrent pairing, such that a stimulus in one modality, e.g., the letter “A” – the inducer, reliably elicits an experience in another modality, e.g., the color red – the concurrent, then ASMR can reasonably be characterized as a synesthetic-type response. Audio, visual, tactile, and social stimuli termed “ASMR triggers,” e.g., whispering, soft hand movements, can be thought of as inducers that elicit concurrent tactile sensations (pleasant tingling at the scalp) and feelings of relaxation. Indeed, early theorizing suggested that the experience of ASMR may be a form of auditory-somatosensory synesthesia (Poerio, 2016) or auditory-emotion synesthesia in which the tingling sensation is not a primary concurrent, but rather a secondary phenomenon (Barratt and Davis, 2015).

Subsequent theoretical accounts of ASMR have gone further in proposing that ASMR occurs specifically as a result of cross-activation between the primary auditory cortex and regions underlying affective touch in the insula (McGeoch and Rouw, 2020). This synesthetic insula cross-activation *via* auditory cortex modulates regulation of sympathovagal balance, pushing it toward the parasympathetic side, thus accounting for positive effects of ASMR triggers on emotion *via* the autonomic nervous system. Although theoretically driven, the model of McGeoch and Rouw (2020) is supported by evidence demonstrating that state ASMR is associated with reductions in heart rate (Poerio et al., 2018) and increased activity in somatosensory, motor, visual, and auditory cortices (Lochte et al., 2018; Smith et al., 2019) and more recently, evidence of heighted interoceptive sensitivity in ASMR-responders compared to controls (Poerio et al., 2022).

Other research suggests that ASMR and synesthesia share a broader neurocognitive phenotype. For instance, both ASMR-responders and synesthetes (compared to controls) typically score higher in the personality trait of openness to experience, lower in the personality trait of conscientiousness, and higher on the fantasizing subscale of the interpersonal reactivity index, an index of empathetic responding (Rouw and Scholte, 2016; McErlean and Banissy, 2017; Fredborg et al., 2018). Both are also associated with altered patterns of resting-state functional connectivity in large-scale neural networks. Synesthetes shows heightened functional connectivity within and between networks (for reviews see Hubbard et al., 2011; Rouw et al., 2011). For instance, grapheme–color synesthetes show significantly stronger intrinsic functional connectivity within seven intrinsic connectivity networks compared to controls (Hubbard et al., 2011; Rouw et al., 2011; van Leeuwen et al., 2011; Dovern et al., 2012). ASMR-responders, however, show patterns of reduced within-network connectivity, but heightened between-network connectivity, including between the default mode network and executive and visual resting state networks (Smith et al., 2017, 2019). Although intriguing, these parallels do not unequivocally demonstrate a relationship between ASMR and synesthesia. Indeed, the same findings could equally, and perhaps more convincingly, be used to draw parallels to more prevalent phenomena under the umbrella term of “aesthetic experiences” such as chills, elevation, awe, or feeling moved (Haidt, 2003; Grewe et al., 2011; Menninghaus et al., 2015). For example, the openness-to-experience trait is strongly associated with the propensity to experience aesthetic chills (Nusbaum and Silvia, 2010), and more empathetic individuals are more likely to experience chills associated with feeling moved (Bannister, 2019). Similarly, patterns of resting-state functional connectivity in ASMR are arguably more comparable to patterns observed in aesthetic experiences: a greater propensity for aesthetic chills is related to increased functional connectivity between the default mode network and sensory and motor regions (Williams et al., 2018).

Only two studies have examined the presence of synesthesia in ASMR responders, which provides more compelling support for the connection between the two cross-modal experiences. Barratt and Davis (2015) reported a synesthesia prevalence rate of 5.9% in their ASMR sample, with 29 participants reporting several synesthetic mappings, including grapheme–color, time–space, and pain-gustatory. This study assessed synesthesia by providing a description and asked participants to report whether they had any type of synesthesia with a single question (“Do you have any type of synesthesia?”). However, compared to a general population prevalence rate of 4.4% (Simner et al., 2006), the prevalence of synesthesia in their ASMR sample was only marginally higher (*p* = 0.06). Rouw and Erfanian (2018) found a significant correlation between ASMR and reporting “other” self-described types of synesthesia, but no relationship between ASMR and reporting sequence-color, sequence-shape, or hearing-color subtypes. The existing evidence, therefore, appears to show only a weak link between ASMR and (some types of) synesthesia.

Comparing the incidence of synesthesia in an ASMR sample to population estimates of synesthesia is problematic for several reasons. Such a comparison will produce different conclusions depending on the comparison rate chosen. Synesthesia prevalence rates are highly variable with some far higher than 4.4% (Chun and Hupé, 2013; Rouw and Scholte, 2016; Rouw and Erfanian, 2018) and others much lower (Baron-Cohen et al., 1996). Direct comparisons to previous prevalence rates are also challenging because they may differ from the study in the types of synesthesia captured, the assessment methods used, and how inclusion criteria are set and applied [see Table 1 in Johnson et al. (2013)]. Finally, and perhaps most importantly, existing population estimates are likely to include ASMR-responders, resulting in a contaminated sample unsuitable for comparison. Although we do not definitively know the population prevalence of ASMR, some estimates are as high as 47% in an undergraduate sample (McErlean and Banissy, 2018), suggesting that this may be a substantial issue. In this study, we overcome these limitations by assessing synesthesia and its subtypes in a large sample of ASMR-responders and non-responders. Doing so allows us to directly test whether the prevalence rates of synesthesia are indeed significantly higher in ASMR-responders compared to non-responders, a finding that may be indicative of common genetic or neural mechanisms.

**Table 1:** Prevalence estimates of synesthesia subtypes and mirror-touch.

	All participants (*n* = 648)	ASMR responders (*n* = 152)
Any synesthesia	64 (9.4%)	33 (21.7%)
1. Grapheme-color	17 (2.5%)	4 (2.6%)
2. Temporal-color	3 (0.4%)	3 (2.0%)
3. Sequence-space	1 (0.1%)	1 (0.7%)
4. Grapheme-personification	18 (2.6%)	8 (5.3%)
5. Person-color	14 (2.0%)	10 (6.6%)
6. Audition-color/form	12 (1.8%)	6 (3.9%)
7. Tactile-color/form	3 (0.4%)	1 (0.7%)
8. Mirror-touch	37 (5.7%)	16 (10.5%)

## Materials and methods

### Participants

We recruited 648 participants (276 males, 371 females, and 1 other; mean ± SD age = 33.0 ± 14.4 years, range 18–60 years), comprising 247 college students and 401 working adults. A short oral presentation on the research was given to 361 college students in the classroom, after which 68.4% of them completed the online survey in class. Working adults were randomly recruited by a temp agency (Agekke Corp, Tokyo, Japan) with a short description of the research. Of the total sample, 243 respondents indicated that they experienced or watched ASMR content online (*n* = 174, 70% in the student sample; *n* = 69, 17% in the working adult sample). The study was approved by the Research Ethics Committee of Chukyo University (approval no. RS20-013). Experimental procedures were implemented in accordance with Ethical Guidelines for Medical and Biological Research Involving Human Subjects. All participants were informed of the purpose of the study. The online survey was anonymous and informed consent was obtained from all the participants.

### Measures

Participants completed a 10-min survey comprising a synesthesia questionnaire (Chun and Hupé, 2013) and an ASMR questionnaire (Barratt and Davis, 2015). The order of the questionnaires was fixed. The survey was conducted using Google forms between July 2020 and July 2021.

As per Chun and Hupé (2013), the synesthesia questionnaire consisted of questions with yes/no responses to indicate experience with seven types of synesthesia: (1) grapheme–color (letters and/or numbers evoking colors/forms), (2) temporal color (numbers and/or time sequences evoking colors/forms), (3) sequence-space (numbers and/or time sequences organized in space), (4) grapheme-personification (letters and/or numbers associated with gender/personality), (5) person-color (colors associated with people), (6) audition-color/form (sounds/voices/music evoking colors/forms), and (7) tactile-color/form (touch evoking colors/forms). (8) The following question about mirror touch was also asked: “When you observe a person being touched on a place on his/her body by someone, do you feel the sensation on your own body on the place where the person was touched?” If participants answered any of the questions affirmatively, they were asked to explain their experience and were encouraged to give examples. These detailed descriptions were later used to determine whether participant descriptions aligned with veridical synesthetic experiences or reflected other cultural or metaphorical associations. Although self-reported synesthetic mappings fall short of gold standard diagnostics for synesthesia (e.g., consistency tests), self-reported synesthesia has been corroborated by tests of genuineness. For example, of 32 self-referred synesthetes (colored-word and colored-music), 26 (81%) met criteria for genuineness after testing (Baron-Cohen et al., 1996). For ASMR, participants first indicated whether they had ever watched ASMR videos. If they answered affirmatively, then they were asked several questions about their engagement with online ASMR content, e.g., time of day when ASMR videos are watched, and their experience of ASMR, e.g., the presence and location of tingling sensations. To provide an index of the strength of the ASMR response, ASMR participants also completed a measure of flow to ASMR experiences (Barratt and Davis, 2015) that comprised 8-items assessing the extent to which their engagement with ASMR content elicited a flow-like mental state, e.g., “My attention is focused on what I am feeling” or “I feel totally in control.” Responses were made on 5-point Likert scales from 1 (not at all) to 5 (completely), which were summed to provide an overall flow-to-ASMR score (range 8–40) such that higher scores indicated a greater propensity to experience absorption and immersion when engaging with ASMR content. Previous research shows that flow-to-ASMR is linked to ASMR propensity as measured by the ASMR-15 (Roberts et al., 2019).

### Data analyses

We checked the distribution of flow-to-ASMR scores: sample size 243; range 8–40; skewness-0.156; kurtosis 0.986. The Shapiro–Wilk test indicated that the data did not follow a normal distribution: *W* = 0.981, *p* = 0.003. Data from three participants were more than 3 SDs away from the mean, but the Grubbs test did not reveal any outliers in the data (*p* > 0.10). Thus, we used all data (*N* = 243) for subsequent analyses involving this scale. The Kaiser–Meyer–Olkin (KMO) measure of sampling adequacy was assessed on the basis of partial correlation coefficients between the items. The KMO value was 0.73 so that the sample size was considered reasonable. In general, the cutoff value is set at 0.50. Previous research has demonstrated that a single factor best captures the flow-to-ASMR questionnaire (Barratt and Davis, 2015). Using the maximum likelihood estimation, we also confirmed that a one-factor solution was optimal (eigenfactor = 2.99). The one-factor model accounted for 37.4% of the variance in flow-to-ASMR scores. Factor loadings for the eight items were greater than 0.40 (range 0.41–0.62). Therefore, we conclude that the ASMR score adequately captures participant flow-like ASMR experience.

We performed cross-tabulation analyses to examine relationships between ASMR responders and synesthetes. These variables were derived from categorical data so that we used Fisher’s exact test for subgroup comparisons. Pearson’s χ^2^ statistic and Cramér’s phi were computed in 2 × 2 or 2 × 3 contingency tables. Cramér’s phi is a measure of association between two variables, giving a value between 0 and 1 (inclusive).

To test for the existence of distinct groups sensitive to ASMR (Swart et al., 2022), we performed latent class analyses on the flow-to-ASMR questionnaire. We determined the optimal number of classes using the following criteria: Akaike’s information criterion (AIC), adjusted Bayesian information criterion (BIC), adjusted Lo–Mendell–Rubin (LMR) likelihood ratio, and entropy. The AIC and adjusted BIC measure the complexity of an evaluated model in terms of degrees of freedom and penalizes more complex models. Lower AIC and adjusted BIC values reflect a better fit to the data. The adjusted LMR compares the fit of the specified class solution to a model with one fewer class. A significant value of *p* suggests that the specified model provides a better fit to the data. Entropy refers to the confidence with which individuals can be classified into different classes. A higher value indicates a clear delineation of membership.

We used R^1^ and IBM SPSS Statistics (version 25) to analyze these data. Latent class analysis was performed using Mplus (version 8.7). All datasets are available from Supplementary Material.

## Results

### Prevalence of synesthesia and subtypes

We estimated the prevalence of synesthesia and subtypes (see Table 1). In our sample (*N* = 648), 64 participants reported at least one type of synesthetic association, corresponding to a prevalence rate of 9.9%. Grapheme–color, grapheme-personification, and person-color subtypes were the most commonly reported (2.6, 2.8, and 2.2%, respectively); temporal-color, tactile-color, and sequence-space^2^ were the least commonly reported (0.5, 0.5, and 0.2%, respectively). Synesthetic associations were significantly more prevalent in females (13.8%) than in males (4.7%): Fisher’s exact test, *p* < 0.001.

### Prevalence and intensity of ASMR

**Figure 1:**
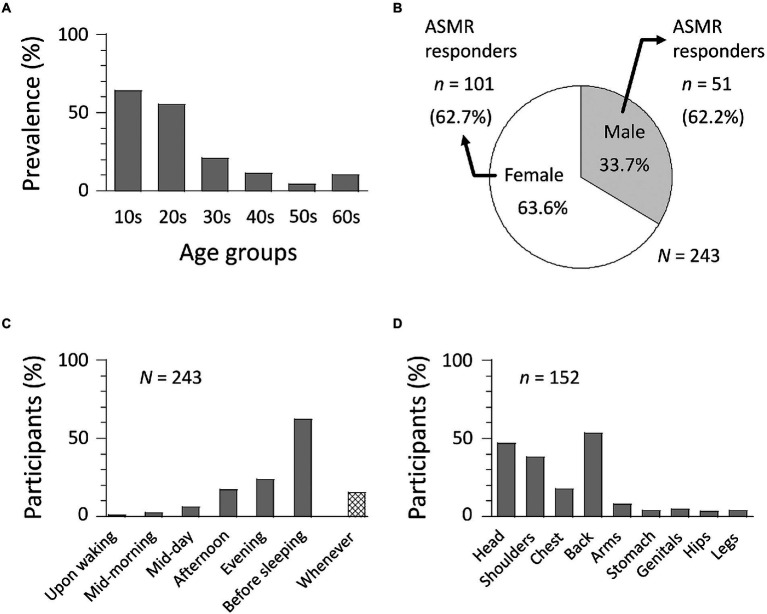
Results of an online survey about ASMR experiences (*N* = 648). **(A)** Percent of participants who watch ASMR videos (*N* = 243) across age groups. Note that 10s refers to participants in the teens (i.e., 18 and 19 years old). **(B)** Gender differences in ASMR experience. ASMR responders (*n* = 152) are those who indicate tingling sensations when watching ASMR videos. **(C)** Time of day at which ASMR videos were watched. **(D)** Anatomical locations of ASMR tingling sensations. Multiple answers were allowed in panels **C**,**D**.

Of all participants (*N* = 648), 243 people (37.5%) reported watching ASMR content online. This was substantially higher in the student sample (*n* = 174, 70.4%) than the working adult sample (*n* = 69, 17.2%) (Fisher’s exact test, *p* < 0.001), likely due to their greater awareness of ASMR content online. Indeed, more than half of teenagers (63.9%) and participants in their twenties (55.3%) reported watching ASMR videos, whereas the proportion of participants over 30 years old was only 11.8% (see Figure 1A). Reports of watching ASMR videos were also greater among females (63.6%) than males (36.4%): Fisher’s exact test, *p* < 0.001 (Figure 1B) and participants tended to engage with ASMR content primarily before sleeping (61.1%, Figure 1C).

Since watching ASMR content online is not sufficient to experience ASMR *sensations*, participants were asked about their experiences of watching ASMR to determine ASMR responder status. Previous research suggests that a canonical feature of ASMR is the presence of tingling sensations in the head, back, and shoulders, in response to ASMR triggers. We therefore classified participants as ASMR-responders if they reported tingling sensations predominately within the head/shoulders/back regions in response to watching ASMR content. Of 243 participants who reported watching ASMR content, 152 (62.6%) were classified as ASMR responders, giving an overall prevalence rate of 23.5%. There was no gender difference in ASMR responders: 62.2% for males and 62.7% for females: *t* = 0.08, *p* = 0.95, Cohen’s *d* = 0.01 (Figure 1B). ASMR sensations were reported as having been felt predominately in the back of the body (53.3%), head (46.7%), and shoulders (38.2%) (Figure 1D).

**Figure 2:**
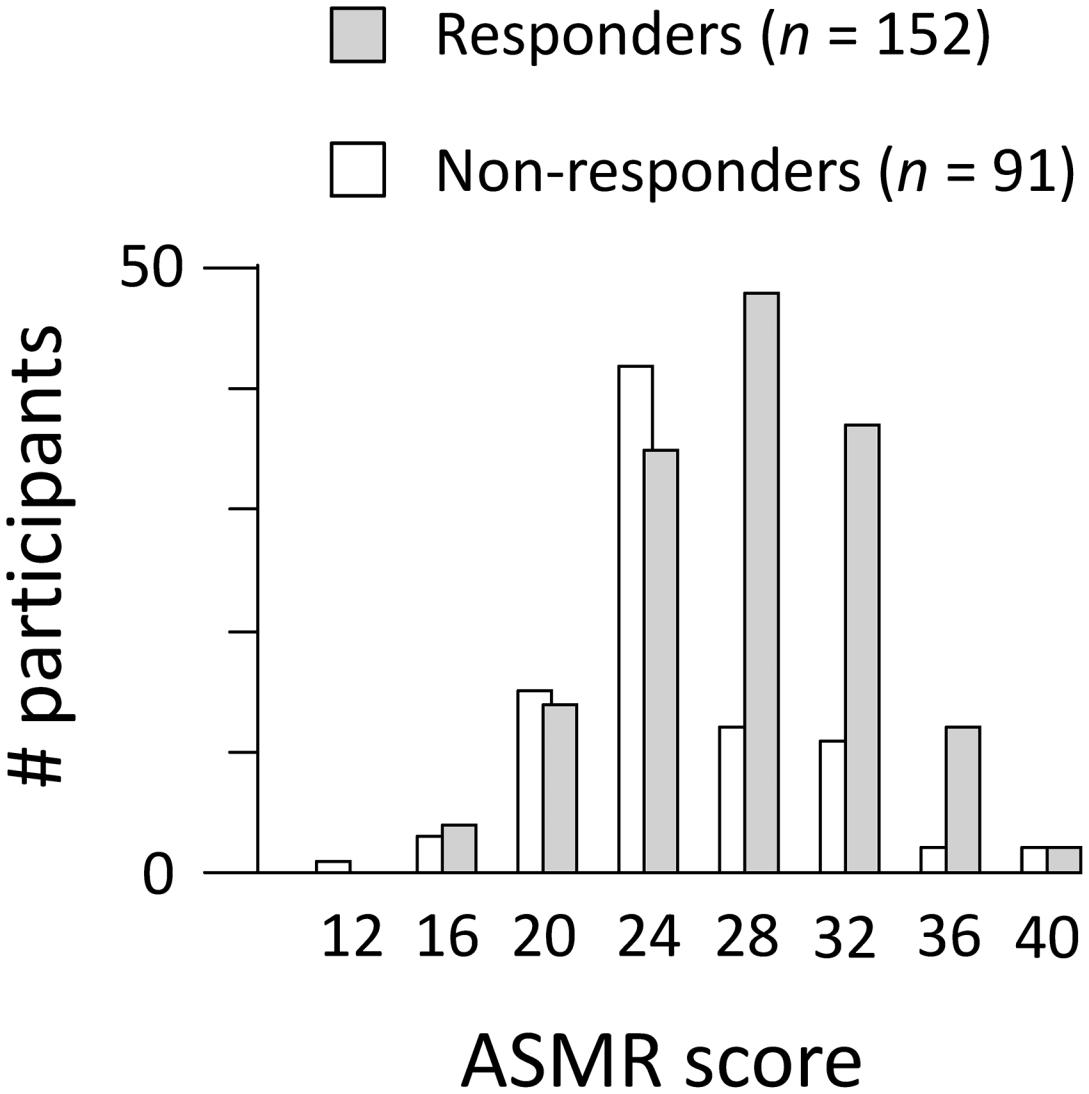
Histogram of ASMR scores (*N* = 243). Participants were classified as ASMR responders or non-responders (see main text). The averaged ASMR score was higher for responders than non-responders.

Next, we examined whether flow-to-ASMR content scores differed between participants who watch ASMR content with and without reported ASMR-tingling sensations, i.e., ASMR responders vs. non-responders who watch ASMR videos. Average flow-to-ASMR scores for the 152 participants classified as ASMR-responders were significantly higher (26.3 ± 4.7) than the 91 non-responders (23.5 ± 5.7): *t* = 4.08, *p* < 0.001, Cohen’s *d* = 0.88 (Figure 2). We also examined differences between ASMR-responders and non-responders on each of the 8 flow-to-ASMR scale items (see Table 2). ASMR-responders had significantly higher scores compared to non-responders on the following four items: “Things seem to happen automatically,” “My attention is focused on what I am feeling,” “It is no effort to keep my mind on what is happening,” and “I am not worried about what people think of me.”

**Table 2:** Individual items endorsed on the flow-to-ASMR questionnaire.

Questionnaire item	Responders (*n* = 152)		Non-responders (*n* = 91)				
	Mean	SD	Mean	SD	*t*-value	Value of *p*	Cohen’s *d*
My attention is focused on what I am watching	3.7	1.0	3.5	1.0	1.38	0.169	0.18
My attention is focused on what I am feeling	4.1	0.9	3.4	1.1	5.18	<0.001	0.68
Time seems to alter	2.9	1.1	2.7	1.2	1.50	0.134	0.20
Things seem to happen automatically	4.1	0.8	3.3	1.2	6.07	<0.001	0.81
It is no effort to keep my mind on what is happening	3.5	1.0	3.0	1.1	3.03	0.003	0.41
I feel totally in control	2.5	1.1	2.3	1.1	1.32	0.190	0.17
Time seems to stop	2.4	1.1	2.5	1.3	−0.44	0.660	0.06
I am not worried about what people think of me	3.2	1.2	2.8	1.2	2.62	0.009	0.35

### Is synesthesia more prevalent among ASMR responders?

Of the 152 ASMR responders, 33 reported at least one type of synesthetic association, corresponding to a 21.7% prevalence rate of synesthesia in ASMR. Of the 91 participants who reported watching ASMR content, but not expecting ASMR-tingling (non-responders), 10 reported at least one type of synesthetic association, corresponding to a prevalence rate of 11.0%. Of the remaining participants who did not experience ASMR, 21 out of 405 reported at least one type of synesthetic association, corresponding to a prevalence rate of 5.2%. Taken together, these results suggest that over half of those identifying as synesthetes also experienced ASMR, and that synesthesia is at least twice as common among ASMR-responders compared to non-responders (who watch ASMR content) and four times as common among ASMR-responders compared to those who do not watch ASMR content (see Table 3 for the cross tabulation of ASMR-responder, ASMR non-responder and “Other” by synesthetes).

**Table 3:** Numbers of participants categorized as ASMR responders and synesthetes.

	ASMR		Others (*n* = 405)	Total sample
	Responders (*n* = 152)	Non-responders (*n* = 91)		
Synesthetes	33	10	21	64
Row percent	51.6%	15.6%	32.8%	9.9%
Adjusted residual	5.59*	0.38	−5.17*	n/a
Non-synesthetes	119	81	384	584
Row percent	20.4%	13.9%	65.8%	90.1%
Adjusted residual	−5.59*	−0.38	5.17*	n/a
Column total	152	91	405	648
Column percent	23.5%	14.0%	62.5%	100.0%

The results indicate that sensitivity to ASMR stimuli inducing a tingling sensation is associated with synesthesia: Pearson’s χ^2^(1) = 34.06, *p* < 0.001; Cramér’s phi = 0.23, *p* < 0.001. Adjusted residuals indicate the difference between actual and expected counts relative to sample size. *p < 0.05, Bonferroni correction for multiple comparison.

**Figure 3:**
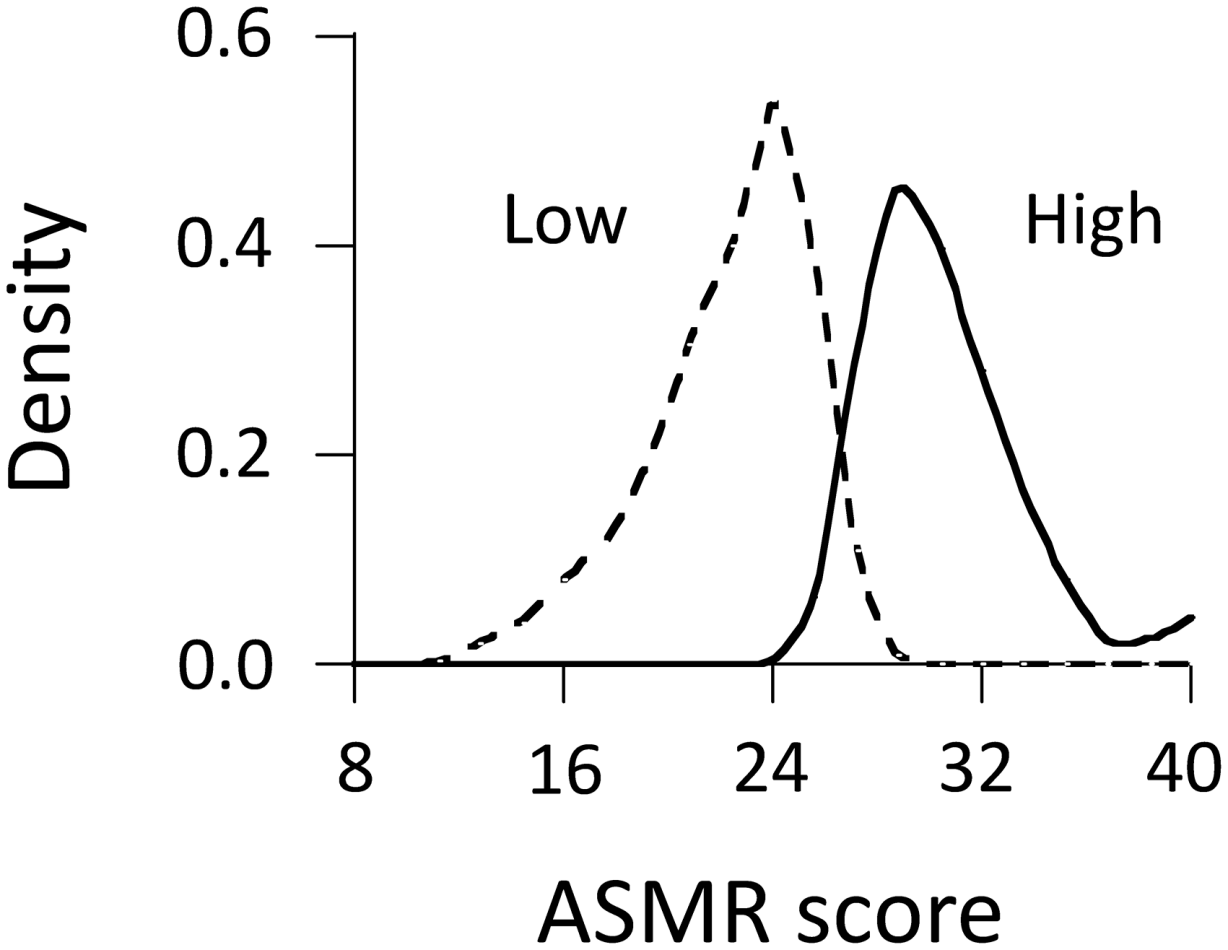
Distribution of flow-to-ASMR scores (*N* = 243). High-and low-score groups comprised 90 and 153 participants (37.0 and 63.0%), respectively. The high-score group included 68 ASMR responders (75.6%), whereas low-score groups included 84 ASMR responders (54.9%).

We performed latent class analyses to look for a best-fitting model for quantitative data of flow-to-ASMR scores (Table 4). A two-class solution yielded a significant adjusted LMR (*p* < 0.001), compared to a one-class solution. Entropy (0.72) was adequate in a two-class model. AIC and adjusted BIC values of a three-class solution were lower than those of two-class solution. However, adjusted LMR did not reach statistical significance (*p* = 0.15). The four-class model was rejected for the same reason (*p* = 0.42), although AIC and adjusted BIC values decreased from the three-class model to the four-class model. From the view of parsimony, we determined that the two-class model fit the data best. Class membership of this model indicated that 37.0 and 63.0% of 243 participants belonged to high-and low-score ASMR groups, respectively (Figure 3). The proportion of the high-score group differed from that of ASMR responders (62.6% of the participants). Table 5 shows a cross tabulation on ASMR score groups by synesthetes. The high-score ASMR group was not associated with synesthetes: Fisher’s exact test, *p* = 0.45. This suggests that subjective intensity of flow-to-ASMR scores is not linked with synesthesia.

**Table 4:** Latent class analysis based on ASMR scores (*N* = 243).

Class	AIC	adj. BIC	adj. LMR	*p*	Entropy
One	5,827	5,832	n/a	n/a	n/a
Two	5,625	5,633	214.98	<0.001	0.72
Three	5,547	5,558	93.99	0.15	0.81
Four	5,533	5,547	32.09	0.42	0.84

AIC, Akaike’s information criterion; BIC, Bayesian information criterion; LMA, Lo–Mendell–Rubin likelihood ratio.

**Table 5:** Numbers of participants in ASMR score groups and synesthetes.

	ASMR		Total percent
	High-score group	Low-score group	
Synesthetes	15	28	43
Row percent	34.9%	65.1%	17.7%
Adjusted residual	−0.32	0.32	n/a
Non-synesthetes	75	125	200
Row percent	37.5%	62.5%	82.3%
Adjusted residual	0.32	−0.32	n/a
Column total	90	153	243
Column percent	37.0%	63.0%	100.0%

Pearson’s χ^2^(1) = 0.10, *p* = 0.75; Cramér’s phi = 0.02, *p* = 0.75.

## Discussion

Autonomous sensory meridian response is a complex sensory-emotional experience characterized by relaxing tingling sensations originating in the scalp. It is a feeling elicited in some people by stimuli including whispering, soft touch, personal attention, crisp sounds, and slow hand movements. Since the term “ASMR” was coined, it has attracted attention from psychological science. ASMR has been likened to synesthesia, with parallels between the two inferred by their similar phenomenology and neurocognitive profiles. However, *empirical* evidence directly linking ASMR with synesthesia is sparse and appears to show only a weak link between ASMR and some types of synesthesia (Barratt and Davis, 2015; Rouw and Erfanian, 2018). In this study, we examined, for the first time, whether the prevalence of synesthesia is indeed significantly higher in ASMR-responders compared to non-responders. Such an association would provide empirical support for theoretical accounts of ASMR that are based on mechanisms thought to underlie synesthesia (McGeoch and Rouw, 2020).

In the present study, a large sample of working adults and students (*N* = 648) was surveyed about their experience with ASMR and common types of synesthesia. Of the whole sample, 9% reported at least one type of synesthetic mapping and 23% were classified as ASMR-responders. An additional 14% watched ASMR content online, but did not report feeling the canonical ASMR-tingling, and were therefore classified as non-responders. At 9%, our overall prevalence rate of synesthesia is comparable, but typically lower than rates observed in studies using similar methodology, where self-reporting is sufficient for inclusion, e.g., 19% for Chun and Hupé, 2013, 9–17% for Rouw and Erfanian, 2018, 24% for Rouw and Scholte, 2016, 16–23% for Barnett et al., 2008). After removing ASMR-responders, synesthesia prevalence was 5%, a rate more consistent with studies using more stringent inclusion criteria (Simner et al., 2006).

To our knowledge, there are no previously reported prevalence rates for ASMR. However, one study, which did not use additional verification methods for ASMR-responder classification as was done here, suggested a 47% prevalence rate in a student sample (McErlean and Banissy, 2018). Based on the current findings we would tentatively suggest a prevalence rate for ASMR of approximately 20%, i.e., around one in five people experience ASMR. This would, of course, need to be verified with appropriate methodology, ideally with random sampling across demographics, and with additional verification measures for ASMR responses (Swart et al., 2022) and consistency (Poerio et al., in preparation). Of course, some participants in the present study may not be genuine ASMR-responders so our prevalence rate could be an overestimate; it could also be an underestimate because there may have been people who experienced ASMR sensations without being familiar with the terms ASMR or ASMR content online. Our central research question concerned the co-occurrence of ASMR and synesthesia – is synesthesia more common among ASMR-responders, and is ASMR more common among synesthetes? The answer to both, it would seem, is yes. In our sample, 52% of synesthetes were classified as ASMR-responders. The proportion of ASMR-responders who were also synesthetes was 22%. Taken together, these results suggest that: (1) over half of those identifying as synesthetes also experience ASMR, and (2) that synesthesia is at least twice as common in ASMR-responders as in non-responders who watch ASMR content (22% vs. 11%) and four times as common as among those who do not watch ASMR content at all (22% vs. 5%).

The rate of synesthesia among our ASMR-responders (22%) was nearly four times higher than reported in Barratt and Davis (2015), who reported a 5.9% incidence of synesthesia in ASMR. Twenty-eight of their 33 cases were deemed genuine after asking for descriptions of inducer-concurrent mappings (see their Supplementary Material) and comprised more mappings than we asked about in the present investigation, e.g., music-form, sound-taste. One possibility for the discrepancy between studies in synesthesia prevalence in ASMR is the method of assessment, which may have led to an underestimation of synesthesia in the former study and an overestimation of synesthesia in the current study.

Barratt and Davis (2015) assessed synesthesia by providing a description (“perception in one sense triggering sensation in another, unstimulated sense. For example, you may “see” the letters as having colors, or sense shapes from music”) and asked participants to report whether they had any type of synesthesia with a single question (“Do you have any type of synesthesia?”). In our study however, we made no mention of synesthesia, and instead asked a series of questions intended to tap specific inducer-concurrent mappings, e.g., “Do you associate letters or numbers with specific colors?” One possible explanation is that due to the method used, many more ASMR-responders in the Barratt and Davis (2015) study *did* experience synesthetic-type mappings, but did not report having synesthesia due to unfamiliarity with the term and/or unawareness that their cross-modal correspondences are in any sense remarkable. Similarly, our method may have *overestimated* the prevalence of genuine cases of synesthesia because we did not explicitly examine the consistency or specificity of cross-modal correspondences reported against hallmarks considered necessary for canonical synesthesia (Deroy and Spence, 2013). Our results should therefore be considered with caution and followed up by more extensive testing to determine the veracity of self-reported synesthesia (ideally consistency tests) against predefined “diagnostic” criteria (Colizoli et al., 2014).

Irrespective of the limitation of assessing synesthesia through self-reporting, a substantial strength of our study was the inclusion of a non-ASMR sample, because it enabled a direct comparison of synesthesia rates in ASMR-responders and non-responders. Such an “uncontaminated” comparison population is not possible when comparing against existing synesthesia population rates, which include ASMR participants. An additional strength of our method was the use of verification procedures for classifying ASMR-responders, rather than simply relying on self-disclosure. We used experience of ASMR content online as a useful heuristic for initially identifying ASMR-responder status. Of the 243 participants who had watched ASMR content, only 63% were classified as ASMR-responders by reporting the presence and anatomical location of ASMR-tingling. This classification was further supported by an examination of “flow-to-ASMR” scale responses that were substantially higher among our verified ASMR-responders compared to those who watch ASMR content, but do not experience ASMR-tingling. Although it is still a matter of debate whether tingling sensations and location (focused in the upper body) are necessary conditions for trait or state ASMR, recent work suggests that these features distinguish both ASMR-responders from non-responders, and ASMR-responders from false-positives (Swart et al., 2022).

The ability to screen out participants who may engage with ASMR content in the absence of ASMR sensations is vital, given the increasing popularity of ASMR and the widespread use of ASMR triggers/style in popular culture and media (Poerio, 2020). We wish to point out that “ASMR content” is often used synonymously with “ASMR” as a specific sensation/emotional experience, but the two should not be conflated. Watching ASMR or being familiar with the term does not mean that an individual experiences ASMR as a sensation. Greater awareness of ASMR as a term increases the need for more rigorous identification of genuine cases of ASMR, rather than those that simply recognize the term, have seen ASMR content, or have a strong emotional response to ASMR content/triggers that would not be considered state ASMR, i.e., pleasant, calming, upper body orientated tingling in response to specific triggers, but might more closely resemble other experiences such as frisson or misophonia (Koumura et al., 2021; Tada et al., 2022). Similarly, not engaging with ASMR content or not being aware of the term, despite its popularity, does not preclude an individual from experiencing the sensation and being a genuine ASMR-responder. This means that in our study, by first asking participants to indicate their experience with ASMR content online, we may have inadvertently miscategorized genuine ASMR-responders as non-responders. Therefore, it is possible that we have underestimated the prevalence of ASMR-responders in our sample and also, by extension, the number of ASMR-responders with synesthetic mappings.

What might explain the co-occurrence of ASMR and synesthesia? One possibility we discussed in the Introduction is that they share a common genetic and or neurocognitive basis. Support for this idea comes from studies that show heightened sensory sensitivity in both ASMR (Poerio et al., 2022) and synesthesia (Ward et al., 2017), altered patterns of neural connectivity (Dovern et al., 2012; Smith et al., 2017, 2019), developmental origins (Simner and Bain, 2013; Poerio et al., 2018), and a shared broader phenotype (Rouw and Scholte, 2016; Fredborg et al., 2018). Another possibility that may explain their co-occurrence is that people with ASMR and synesthesia are both simply more likely to report anomalous experiences. Although this should be tested more directly, recent research suggests that ASMR-responders are not more likely to self-report unusual sensory experiences compared to a control group (Palmer-Cooper et al., 2021). A third possibility is that ASMR is *itself* an as yet unclassified form of synesthesia that should be added to existing typologies. Although this is a tempting possibility, we would like to conclude by highlighting the ways in which ASMR and synesthesia are different.

First, ASMR inducer-concurrent pairings are less idiosyncratic. Although ASMR-responders show subtle differences in ASMR trigger preferences, studies demonstrate consistency in the ASMR triggers endorsed, e.g., whispering, soft touch, close personal attention, and in the described concurrent location of tingling (Poerio et al., 2018; Swart et al., 2022). Thus, the stimuli that induce ASMR are remarkably similar among responders, unlike synesthesia, in which inducer-concurrent pairings appear to be highly specific to individuals (Martino and Marks, 2001).

Second, rather than traditional one-to-one inducer concurrent mappings specific to each synesthete (Palmeri et al., 2002), ASMR responders typically have what we might call a many-to-one inducer-concurrent mapping. Many triggers/inducers induce *the same concurrent* among responders, with the experience of ASMR often occurring with greater intensity when triggers from multiple senses are integrated. In this way, the ASMR inducer is not typically unimodal (Poerio et al., in preparation).

Third, at least for some ASMR triggers, the relationship between inducer and concurrent is not arbitrary. Associating close personal attention, soft touch, slow movements or whispering with feelings of relaxation and pleasant tingling appears to mirror typical experiences of intimacy in close personal relationships (Morrison, 2016). This stands in contrast to synesthetic mappings, which appear to have an arbitrary association that is not immediately explainable (Deroy and Spence, 2013).

Fourth, whereas synesthetic associations are often considered involuntary or automatic responses (Ramachandran and Hubbard, 2003; Ward, 2013), ASMR appears to be more affected by contextual factors, e.g., eating sounds may trigger ASMR in one context, but misophonia in another and susceptible to habitation (termed ASMR immunity). Taken together, these differences suggest that ASMR differs from synesthesia in a number of important and interesting ways. We tentatively suggest that ASMR may best be considered a heightened or exaggerated cross-modal correspondence related to hedonic touch (Spence, 2022), rather than a subtype of synesthesia. Nevertheless, future research would benefit from exploring features that differentiate ASMR from synesthesia, and not only their similarities.

## Data availability statement

The original contributions presented in the study are included in the article/Supplementary material, further inquiries can be directed to the corresponding author.

## Ethics statement

The studies involving human participants were reviewed and approved by the Research Ethics Committee of Chukyo University. The patients/participants provided their written informed consent to participate in this study.

## Author contributions

GP and HK: conceptualization, resources, and writing— review and editing. MU and HK: data curation, formal analysis, and investigation. HK: methodology, project administration, and supervision. GP, MU, and HK: writing—original draft. All authors contributed to the article and approved the submitted version.

## Funding

This study was supported by JSPSKAKENHI (grant nos. 20H01789 and 22 K18659 to HK).

## Conflict of interest

The authors declare that the research was conducted in the absence of any commercial or financial relationships that could be construed as a potential conflict of interest.

## Publisher’s note

All claims expressed in this article are solely those of the authors and do not necessarily represent those of their affiliated organizations, or those of the publisher, the editors and the reviewers. Any product that may be evaluated in this article, or claim that may be made by its manufacturer, is not guaranteed or endorsed by the publisher.

## Supplementary material

The Supplementary material for this article can be found online at: https://www.frontiersin.org/articles/10.3389/fpsyg.2022.990565/full#supplementary-material

## Footnotes

^1^ https://www.r-project.org/

^2^Notably the prevalence of sequence-space associations in our sample was substantially lower than other studies with European samples which suggest a prevalence of between 8 and 13% (e.g., Chun and Hupe, 2013; Ward et al., 2018). One intriguing possibility is that there are cross-cultural differences in the prevalence of sequence-space synesthesia, perhaps due to mental representation of time.
